# Integration of Multiplied Omics, a Step Forward in Systematic Dairy Research

**DOI:** 10.3390/metabo12030225

**Published:** 2022-03-04

**Authors:** Yingkun Zhu, Dengpan Bu, Lu Ma

**Affiliations:** 1 State Key Laboratory of Animal Nutrition, Institute of Animal Sciences, Chinese Academy of Agricultural Sciences, Beijing 100193, China; Yingkun.zhu@ucdconnect.ie; 2 School of Agriculture & Food Science, University College Dublin, Belfield, D04 V1W8 Dublin, Ireland; 3 Joint Laboratory on Integrated Crop-Tree-Livestock Systems of the Chinese Academy of Agricultural Sciences (CAAS), Ethiopian Institute of Agricultural Research (EIAR), and World Agroforestry Center (ICRAF), Beijing 100193, China

**Keywords:** multi-omics, dairy cow, lactation, fertility, metabolic disease

## Abstract

Due to their unique multi-gastric digestion system highly adapted for rumination, dairy livestock has complicated physiology different from monogastric animals. However, the microbiome-based mechanism of the digestion system is congenial for biology approaches. Different omics and their integration have been widely applied in the dairy sciences since the previous decade for investigating their physiology, pathology, and the development of feed and management protocols. The rumen microbiome can digest dietary components into utilizable sugars, proteins, and volatile fatty acids, contributing to the energy intake and feed efficiency of dairy animals, which has become one target of the basis for omics applications in dairy science. Rumen, liver, and mammary gland are also frequently targeted in omics because of their crucial impact on dairy animals’ energy metabolism, production performance, and health status. The application of omics has made outstanding contributions to a more profound understanding of the physiology, etiology, and optimizing the management strategy of dairy animals, while the multi-omics method could draw information of different levels and organs together, providing an unprecedented broad scope on traits of dairy animals. This article reviewed recent omics and multi-omics researches on physiology, feeding, and pathology on dairy animals and also performed the potential of multi-omics on systematic dairy research.

## 1. Introduction

**Figure 1:**
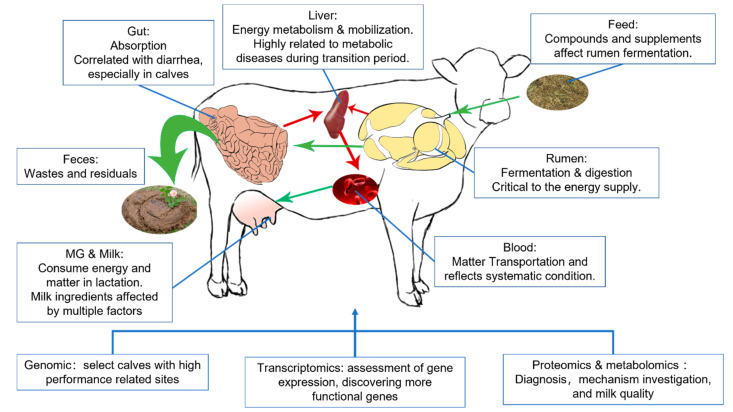
Organs mainly involved in the lactation physiology and utilities of omics. Green and red arrows stand for the nutrition transport and energy supply separately.

Omics, referring to a field of study in biological sciences that ends with -omics, aims at the collective characterization and quantification of pools of biological molecules that translate into the structure, function, and dynamics of an organism or organisms. The development of an automatic DNA sequencer in the early 1990s made whole-genome sequencing possible [1], announcing the dawn of omics. In the following 30 years, novel omics assays have been set up once a corresponding high-throughput qualifying or quantifying method has been established, such as transcriptomics, proteomics, or metabolomics [2,3]. Transcriptomic could clarify and quantify RNA sequences in the sample, representing a snapshot of cellular metabolism, while proteomic and metabolomic divided by chromatography, then qualifying them by comparing mass spectrometry (MS) or nuclear magnetic resonance (NMR) data with databases to capture the function status of target tissues. Omics methods provide researchers with an expanded vision of all detectable molecules on a certain level. They have become an effective multifunctional tool that has been applied from screening differential molecules to sorting phenotypes [4,5,6]. Indeed, single omics provides systematic information on a certain level, but researchers are always eager to have a broader scope. In ruminant research, especially dairy sciences, the factors usually have to pass through more barriers and biology levels than monogastric animals. Those organs are relatively isolated systems while interacting, weaving a tangled web of connection, and making physiology and pathology studies on a single organ or level hard to acquire certain conclusions [7]. In those cases, the multi-omics investigation will promote the exploration of phenotype-related biomarkers and corresponding mechanisms, like how dietary nutrients affect milk components [8] and the impact of rumen microbiota on lactation performance [9]. Current dairy research mainly applies omics methods to breeding, investigating physiology and pathology, developing new traits, and evaluating feed sources and supplements in widely spread organisms from the rumen to spermatozoa. Among those organisms, rumen, liver, and mammary glands are the critical point research spots, which were suggested as critical organs related to the performance of dairy cows [10], in which enriched rumen could ferment dietary carbohydrates into volatile fatty acid (VFA), and also could convert indigestible forage into nutrients by colonized symbiotic microbiota; the liver plays a critical role in processing absorbed nutrient and other bioactive components, acts as the core of fat mobilization, also modifying the component of mammary gland secretion [11]. This rumen–liver–mammary glands network included the path of nutrient molecules transportation from the very first feed intake and ruminant fermentation to finally milk secretion. Different types of omics research on nutrient and metabolic disease have targeted this network, and recent application of multi-omics reported in dairy science focused on relating those organs altogether or discovering the mechanisms of how those organs were affected (Figure 1) [12,13].

However, as the highly specialized symbiotic fermentation system, many omics studies in dairy sciences are integrated with microbiome analysis of the gastrointestinal tract. Genomic and transcriptomics have discovered more traits related to milk production and feed efficiency; non-target proteome and metabolome are with an increase of their significance in the expanding of biomarkers and have contributed to understanding the mechanisms of mastitis and infertility, which could cause massive economic losses to dairy farmers (Table 1).

Even multi-omics applications in the dairy sciences have just started in the recent few years, and it has already become a novel hotspot for research. This article reviewed the application of omics techniques from metagenomics to metabolomics and their integration in the dairy research about lactation physiology, fertility, feeding, management, and diseases, emphasizing the significance of systematic view in the dairy research prospected futural multi-omics utilizations for dairy sciences studies.

## 2. Multi-Omics Studies in Lactation Physiology

Due to their unique multi-gastric digestion system highly adapted for rumination, dairy livestock’s physiology of energy metabolism varied from monogastric animals. Ruminal symbiotic microbes are highly specialized in degrading lignocellulosic biomass into fermentable sugar, finally, fermenting plant-derived carbohydrates into VFAs [63]. Ultimately, VFAs are absorbed through the gastrointestinal tract into the portal vein, utilized by the liver. For dairy animals, rumen, liver, and mammary gland are nodes of the lactation physiology network, corresponding to energy intake, distribution, and output. Because of the vital role of ruminal microbiota in energy metabolism, those organs, with the symbiotic microbiome, provide researchers with a great example of “superorganism” for host–microbial interactions [63].

Rumen and symbiotic microbes directly contribute to rumen metabolites and dietary components and alter the ruminal microbiome by changing fermentation substrates [64]; they are similar to intestinal microbes in monogastric animals in some aspects but have much more impact on the animal body. A high concentrate diet increases the abundance of potentially harmful rumen metabolites like LPS and methylamine in rumen fluid, also higher the risk of rumen acidosis [31]. On the contrary, fresh grass improves microbe colonization, digestion, and microbial protein synthesis and decreases the methane emissions of rumen compared with grass hay [12,35]. Feed composition plays a significant role in shaping the rumen microbiome of calves by modulating initial colonization [65,66]. Proteobacteria is the dominant microbial in the newborn calves, then replaced by Bacteroidetes during the ruminal development [24,67], and ruminants with well-developed ruminal flora are more robust in challenging diarrhea.

Meanwhile, the symbiotic microbiome affects rumen digestion, feed efficiency, and milk production. Cows with different milk yields have significantly altered rumen fluid metabolomic patterns related to protein digestion and absorption, ABC transporters, and unsaturated fatty acid biosynthesis pathways associated with firmicutes, actinobacteria, and synergistetes in the rumen [44]. Metabolomics studies proved that cows with higher feed efficiency showed downregulated amino acid, ruminal linoleic, and alpha-linolenic metabolism [39,45]. Moreover, multiple studies showed that milk performances are related to *Prevotella* [9,46,68]; cows with higher protein yields have a higher abundance of *Prevotella* sp. and lower methane-producing microorganisms in the rumen and related to branched-chain amino acid biosynthesis and less methane emission. Cows with higher feed efficiency might have a microbiome with fewer but more efficient metabolic pathways and dropped low-value metabolites production [22,69,70,71]. For instance, protozoa, a member of the rumen microbiome, have a controversial effect on ruminal digestion [70]. Several studies reported that protozoa are not essential, lead to increased ammonia nitrogen and methane emissions, and negatively correlate to nitro utilization and rumen microbial protein synthesis [72,73]. However, recent research found that protozoa have a positive effect by directly contributing to fiber degradation and indirectly consuming ruminal oxygen to maintain anaerobic conditions, especially in high-forage diets [73,74,75]. Due to the lack of protozoa sequence information, there are still obstacles to discovering its role in ruminal digestion [23]. Even in the same nutritional and management condition, cows also perform different milk yields and milk components related to the rumen microbiome. As demonstrated above, *Prevotella* is correlated with high milk protein yields. Cows with higher saturated fatty acids usually have a higher abundance of lactic acid bacteria (*Lactobacillus*, *Leuconosto*, and *Weissella*) and acetogenic Proteobacteria (*Acetobacter* and *Kozakia*) and showed higher concentrations of butyrate, propionate, and tyrosine and lower concentrations of xanthine and hypoxanthine in the rumen, suggesting those cows might be adapted to reduced rumen pH [51].

The liver is the nexus of lipid metabolism and plays a critical role in ruminant physiology. Nearly 70% of circular glucose in the dairy cow is derived from hepatic gluconeogenesis. After calving, the energy consumption elevates rapidly with the initiation of lactation, meanwhile, decreased dry matter intake (DMI) limited the energy supply, the residual feed intake would soon become negative, leading to the negative energy balance (NEB) [76], in this circumstance, sugar storage will soon be exhausted. Because of maintaining physiological functions, the liver becomes the processing center of mobilized body fat. Ketones derived by triacylglycerol hydrolyzation from adipocytes are usually excessive for liver oxidation, inducing hepatic lipid accumulation, and finally, causing fatty liver and ketosis [77]. These effects also alter the lipid composition of the milk; cows with serum BHB (β-hydroxybutyrate) higher than 0.1 mmol/L may have lower C6 (caproic acid), C22:1ω9 (Erucic acid), C22:5ω3 (Decosapentaenoic acid, DPA), and C23 (Tricosanoic acid) [78]. Varied milk LCFA and VLCFA concentrations also reflect the risk of ketosis and metabolic changes in ewes and donkeys [79,80]. Nearly 50% of dairy cows suffer from metabolic diseases in their first month of lactation [81]. However, it seems that the liver has adapted to the metabolism condition before calving under the regulation of transcript factor PPARA and NFE2L2 [82]. Cows with higher lipid mobilization have an altered plasma lipidome [36,83], indicating other metabolic pathways may also be influenced. L-carnitine showed a potential metabolism to promote the effect of Non-esterified fatty acids (NEFA) and reduce lipid accumulation in the liver [84]. However, the L-carnitine does not seem to affect the hepatic transcriptome profile [19]. Until now, a large number of essential hepatic genes, proteins, and metabolites related to lactation physiology have been reported, but it is still hard to build a systematic view of how liver function affects lactation, the differences between physiological and pathological conditions are also waiting to be investigated by a systematic scope.

Milk, the main product of dairy animals, as mentioned above, is closely related to the condition of rumen and liver. It is widely known that milk yield and components are heritable [43]. Genomic research showed that single nucleotide polymorphisms (SNPs) of *RAP1A* and *DGAT1* are correlated with milk protein yield [85,86]. Transcriptome also revealed that more than 33,000 SNPs are associated with lactation, in which expression levels of 31 genes are directly related to milk yield [18]. Apart from heritable reasons, milk production is directly influenced by dietary structure. Alfalfa hay, rice straw, and corn straw could change mammary glands and liver’s transcript and protein profile [37,43]. However, protein expression changes are sometimes not positively correlated with their corresponding mRNAs [38].

Meanwhile, the rumen microbiome and feed components would change lactation performance. Varied rumen microbiome would induce a different milk fatty acid profile with altered rumen fermentation and protein metabolism under the same diet [51]. Different forage sources could also change the function and composition of the rumen microbiome. Cows fed with corn stover have a significantly lower abundance on gene encoding lactaldehyde reductase, glutamine synthetase type I, methylmalonyl-CoA decarboxylase, succinate dehydrogenase, and alpha-xyloside ABC transporter in the rumen microbiome [43]. Mammary glands are the output positions in lactation physiology, also are the most direct factors related to the milk components and yield. Advancing their knowledge would bring researchers more precise methods for mastitis diagnosis and milk quality assessments.

The rumen–liver–mammary gland network is the core of lactation physiology. Each element, including genotypes, feed components, rumen fermentation, liver conditions, mammary gland function, the interaction between symbiotic microbes and host, also different tissues and organs within the host, would influence the final milk production and milk contents. Even though many metagenomic, transcriptomic, and proteomic studies have been administrated in dairy sciences, there is still not enough information about how different organs are connected within physiological conditions. Studies are focused on the changes induced by specific changes but not on the normal condition. Indeed, due to the individual differences, it is hard to define a “normal condition” of dairy animals, but with the expansion of bioinformatics, a widely accepted baseline multi-omics fingerprint map may be established—leading to a novel recognition of the lactation physiology of dairy animals.

## 3. Multi-Omics Methods for Reproduction Research

The fertility of dairy cows has declined gradually since the 1980s, causing an increased eliminating rate and reduced probable life [87]. Subfertility has become a significant problem with the increasing milk yield of dairy cows and causing tremendous economic losses [88,89]. Genomic research showed that daughter pregnancy rate could become a fertility prediction index [90], and different breeds of dairy cows share few SNPs related to the reproduction traits [16], while bulls have more conservative x chromosomal fertility-related SNPs [91]. However, the clinical evaluation of fertility mainly focuses on morphological features and hormone levels, which are unilateral measures that lack objectivity [82]. Thus, even omics applications in ruminant reproduction are still limited compared with reproduction research in humans, the systematic information acquired by omics approaches becomes significant to expanding our fertility and reproduction knowledge.

Fertility differences in the dairy cattle also performed in the proteome pattern in the gamete. More than 125 proteins significantly differ between bulls with high and low fertility [53]. Those proteins are related to TCA-cycle, ATP concentration, and mitochondria functions [92,93]. Those differences also appear in the transcription level of semen [17]. The expression level of miRNAs also differs in bulls with different fertility and infertility rate [20,94,95]. Pear-shaped sperm is one of the widely known sperm deformity patterns, which has a different protein profile related to reduced antioxidative activities, sperm capacitation, and cytoskeleton [55].

The fertility property of cows is much more complicated compared to bulls. The omics pattern of oocytes is also related to fertility even before ovulation [58]. Even the mechanism is complex. Nevertheless, oxidative stress and inflammation could be the primary reasons for decreased fertility [96]. For instance, annexins, a family of proteins related to anti-inflammation, have higher levels in the uterus fluid in early pregnancy and glutathione-S-transferases concentration during the late estrus [54,97]. Meanwhile, some proteins could perform varied relationships to the fertility in different sections of the reproductive tract, tissue inhibitor of matrix metalloproteinase 2 (TIMP2), an enzyme related to trophoblast invasion and possibly in endometrial remodeling, have an increasing level in uterus fluid with the progress of pregnancy [98]. However, cows with lower fertility have two times higher TIMP2 concentrations in the follicular fluid than regular cows [58], while TIMPs also perform as biomarkers in human reproduction [99]. These may suggest that proteins perform varied functions in the different stages of reproduction. Metabolites in the reproductive tract are also correlated to fertility. Methionine, an amino acid that has been approved that could change rumen microbiota and improve milk components [8], has a different abundance in the follicular fluid between maiden and first parous heifers [33]. Guerreiro et al. [100] discovered the differential metabolites between the follicular fluid in cows with different fertility divided by oocyte production, and found that antioxidative metabolites resveratrol 4′-glucoside, lupinisoflavone N, peonidin acetyl 3,5-diglucoside, 3,3′,4,5′-tetrahydroxy-trans-stilbene, 5,7-dihydroxy-6-methyl-8-prenylflavanone, xanthohumol, and prostaglandin M could become the marker of high fertility.

Omics methods assist researchers in discovering and locating the reproduction-related genes in both cows and bulls, and they also emphasized that oxidation and inflammation are the main factors related to fertility and provide researchers new biomarkers in the screening of high fertility calves. Furthermore, studies that applied the omics approach have expanded our knowledge about the microenvironment reproduction tract and indicated the effect of metabolites on the implantation and pregnancy process. Through the multi-omics methods, researchers may include the reproduction system into the rumen–liver–mammary gland network and develop more intervention protocols to improve the reproduction traits.

## 4. Multi-Omics Assists Feeding and Management

Improving feed efficiency is a long run for the dairy industry, as a complicated phenotype, the efficacy of feed utilization depends on genotypes, ruminal fermentation, and feeding components [14]. Genomic studies showed that feed efficiency is heritable and optimized via genomic selection in large herds [101]. Lactation and growth performance are also regulated by ruminal and hepatic micro RNAs (miRNA, miR). Dietary components and particle size could affect the expression of feed efficiency and rumen function-associated miRNAs [21,25].

Periparturient (or transition) period, from 3 weeks pre-calving until three weeks post-calving, has a critical impact on dairy production [102]. In this period, significant physiological, metabolic, and nutritional changes occur when most metabolic disorders occur [103]. Understanding the physiological, metabolic, and nutritional changes will help to improve current management for the better welfare of periparturient dairy cows and minimize economic losses. The dry period, a regularly 60-day no-milking phase before expected calving to support fetus development and prepare for the next lactation, is widely accepted in dairy farms [104]. On the one hand, a dry-off phase would increase the infection risk of the mammary gland. However, a continuous milking protocol has been described to reduce health problems but reduces milk protein and change proteomic profile, significantly lowering the concentration of colostrum immunoglobulins by nearly 50% and may weaken adequate passive immune transfer [105]. During the dry period, compositions of mammary gland secretions also alter in response to the drying administration, miRNAs related to gestation, lactation, inflammation, and disease formation significantly changed in the different phases of the dry period [27]. The temperature–humidity control is also an essential part of transition period management, cows exposed to high temperature and humidity environment would have heat stress. According to Skibiel et al. [61], heat stress that occurs in the dry period would affect liver proteome profile, interferes oxidative phosphorylation, mitochondrial function, farnesoid X receptor/retinoid X receptor (FXR/RXR) activation, and the methylmalonyl pathways; reduce ATP production, aggravates oxidative stress; and accelerate hepatic triglycerides and cholesterol accumulation. Heat stress leads to higher susceptibility to transition-related diseases; a whole-genome analysis showed that high-producing cows are more susceptible to heat stress. Hsp90 protein binding, zinc ion binding, and gated channel activity pathways are also related to heat stress, and at least three different genomic regions on BTA5, BTA14, and BTA15 chromosomes are strongly associated with milk production under heat stress conditions [15]. By the integration of multi-omics, we can find the mechanism that related to stress in the management process and develop a more effective protocol that provide more economic benefits for dairy farmers and better welfare for dairy animals.

Effect of dietary component is also an essential aspect of feed formulation, and feed material could provide energy and substrates for physiological function. Ametaj et al. [31], the first group using metabolomics to evaluate the effect of dietary components on the rumen microbiome, found that 30% and more barley grain could increase the concentration of potentially harmful rumen metabolites. Changes in rumen metaproteomic also observed by Snelling and Wallace [60], dairy cows fed by a high concentrate diet, protozoa structural proteins will dominate proteome profile of ruminal digesta, and bacterial proteins are mainly glycolysis related proteins. Different forage sources could also alter the metabolites profile of rumen fluid and milk, serum, and urine in dairy cows, and alfalfa hay-fed cows have a higher N efficacy, amino acids metabolism, and milk performance than corn stover fed cows [106]. Different processing of the same material could also change milk protein content: heat-treated soybean meal could induce higher milk α-casein abundance and lower β-casein, α-lactalbumin, and zinc-alpha-2-glycoprotein than solvent-extracted soybean meals [56]. A recent study by Veshkini et al. [62,107] performed that the supplement of FAs would improve metabolism of xenobiotics by cytochrome P450, drug metabolism—cytochrome P450, retinol metabolism, and steroid hormone biosynthesis in the transition period; the addition of polyunsaturated fatty acid (PUFA)-enriched marine microalgae also affects ruminal microbiome and FA profile of milk, improves rumen fermentation, and increases concentrations of PUFA in milk [68].

Furthermore, the functions of bioactive compounds in materials should be considered in feed development. For instance, malt, a high-starch ingredient with a lactation inhibitory effect, might not be a suitable TMR ingredient in the lactation period but could be a functional supplement in the dry period [108]. Thus, omics methods become a powerful tool in evaluating the effect of feed additives on the physiology of dairy animals. Wang et al. [50] found that the 300 g/d of *Perilla frutescens* leaf supplementation could upregulate oleanolic acid and nucleotides in milk while downregulating 2-hydroxycaprylic acid and enriching metabolic pathways such as pyrimidine metabolism and biosynthesis of unsaturated fatty acids in both rumen and milk. Rumen-protected nutrients are processed for avoiding the ruminal degradation and final release in the intestine, which have been widely researched in recent years. Elolimy et al. [42] found that rumen-protected (RP) methionine supplement in late pregnancy cows altered their calves’ fecal microbiome and metabolomic profiles to have better growth performance. Further research by Gu et al. [8] showed that RP methionine could increase ruminal *Acetobacter* and *Saccharofermentan* abundance and elevate milk α-ketoglutaric acid concentration and milk fat may explain the beneficial effect to their offspring. Phenotype research also proved that RP methionine supplements improved the oxidative status of dairy cows [109]. Other RP amino acids also had an effect on the rumen metabolomic profile and lactation performance: RP lysine could improve milk production in corn-fed dairy cows but decreased their oxidative stability [110]; supplement RP arginine to pregnant sheep not only alleviated the nutrient restriction during pregnancy, but also altered the amino acid, carbohydrate, and metabolic pattern in umbilical venous blood of fetus [40]. Apart from amino acids, glucose and alkaloids after the RP process also showed altered digestive parameters: 200 g/d of RP glucose supplement increased rumen bacterial richness and diversity, elevated cellulolytic bacteria abundance, and changed rumen fermentation, increased the concentrations of acetate, propionate, butyrate, and total volatile fatty acid [52]; additional RP betaine increased milk yield and milk protein and influenced pathways related to the synthesis of arginine and cyanoamino acid, also the degradation of proline; however, RP betaine had no significant difference on growth performance comparing with unprotected betaine [47,111].

As mentioned above, the rumen microbiota are related to metabolism, lactation, fertility, and feed utilization. While rumen microbiota also relate to the variation of performance among individual cows under the same feeding and management conditions, Xue et al. [9] found that cows with higher milk protein yield showed different microbial compositions of bacteria and archaea, especially *Prevotella* sp. the altered microbiome pattern also performed in their metabolites, rumen fluid of cows with higher milk and milk protein yield have a higher concentration of amino acids, carboxylic acids, and fatty acid. The changes in rumen microbiota also reflect amino acid (glycine, serine, threonine, alanine, aspartate, glutamate, cysteine, and methionine) metabolism. Further analysis found that rumen microbial components, functions, metabolites, and serum metabolites of the host are all related to the phenotype of milk protein yields. The metabolites of the rumen microbiome and host serum have a similar contribution ratio to the milk protein yields. Although rumen flora plays a crucial part in nearly their performances, it is also the main reason for methane emission [112]. Meta-transcriptomics research demonstrates that bacterial, archaeal, and eukaryotic biomass, methane emission, and VFA concentration increased rapidly in the first hour after feed intake, with corresponding changes of carbohydrate-active enzyme transcripts [26]. Furthermore, this process could be recognized as a phenotype with individual varieties. Those rumen microbiome-related phenotypes may be adjusted or improved by probiotics and direct feed microbial (DFMs). Ogunade et al. [113] found that additional 15 g/d live yeast (*S. cerevisiae*) increases eight cellulolytic bacterial genera while optimizing the utilization of oxygen and lactic acid and inhibits the growth of pathogenic *Salmonella*. Supplementation of 0.1% live *Enterococcus faecium* in dietary could significantly increase the propionate concentration in the rumen fluid while inhibiting the emission of methane, and the dose of *E. faecium* supplement has a different impact on the rumen microbiomes [114]. The effect of complex DFMs with multiple microorganism species and their fermentation products on rumen function and host serum metabolomics is also evaluated by Ogunade et al. [46], although two different DFMs showed varied impacts on the rumen microbiome, both DFMs elevated serum glucose, total VFA, propionate, isovalerate, and valerate concentrations in the rumen also showed similar effects on VFA profile and energy status. The multi-omics combines the responses of different levels and organs, provides a whole vision for the impact of feed components to dairy animals, would become a better way for novel feed sources developments

Omics methods help researchers develop novel feed resources and management protocols, evaluate the effects and mechanisms of feed supplements, and use nutrient interventions to improve milk contents. Meanwhile, those methods assist dairy farmers in adjusting the dietary formula and optimizing management to elevate production efficiency and prevent transition diseases. Furthermore, they provide potential solutions to decrease the emission of greenhouse gas. However, limited by sampling time points and current technologies, omics analysis still could not provide a timely response as a monitoring technique but has already shown the advantages in detecting molecular indicators and predicting functional compounds. With the improvement, multi-omics would become a capable tool for feeding and management assessment and herds’ health observation.

## 5. Multi-Omics Promotes Revealing Dairy Diseases

**Figure 2:**
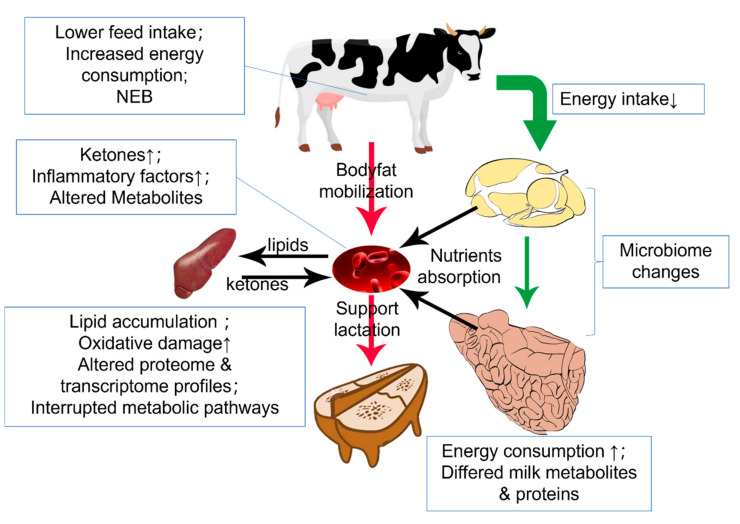
Metabolic condition during the transition period. Down arrow(↓) means decrease and up arrow(↑) means increase. In the transition period, matter intake decreases while the initialed lactation demands more energy. Hence, body fat is mobilized and oxidated into ketone in the liver. The metabolic burden of liver induces oxidative stress and inflammation.

The unique rumen fermentation mechanism allows ruminants to consume plant fiber as a regular diet and degrades indigestible fiber by symbiotic microorganisms [115]. This digest mechanism forms the base of complete digestion and harvesting energy from the ingested feed, making the energy metabolism of ruminants rely on the rumen flora to maintain function. Rumen commensal microbiota reflects the dietary components, and the microbiota metabolites will determine the body’s energy metabolism. Once the fermentation feature is biased from the average level, cows will risk suffering diseases [116,117]. Apart from the rumen, the liver is also a critical organ in metabolic disease, especially in the periparturient period, where NEB usually occurs. Cows in NEB condition will mobilize their body fat into NEFA for β-oxidation in the liver. This process will elevate serum ketone concentrations and hepatic lipid accumulation, directionally leading to ketosis and fatty liver (Figure 2) [118]. At the same time, the different urea cycle and plasma AAs during the late gestation and early lactation may also involve in this process [119]. Researchers have clarified the general etiology of those diseases but still lack systematic knowledge on the molecular level [120].

Without proper feeding and management, energy consumption could finally exhaust glycogen storage during the transition period. To compensate for the negative energy balance, cows mobilize their body fat, and serum adipokine levels are changed to adapt to this process [121]. Therefore, ketones, including NEFA and BHBA (beta-hydroxybutyrate acid), are produced as the intermediate metabolites of lipid metabolization and act as an alternative energy source of glucose. However, this process would interfere with the multiple metabolic pathways and burden the liver. Current research performed that the high concentration of BHB is related to altered serum metabolic profile, anaerobic rumen fermentation, lipid metabolism, and oxidative stress further proved this point [49,79]. Even ketosis and fatty liver are metabolic diseases closely related to lactation physiology, the incidence and severity of those diseases are still heritable [122,123]. Cows with ketosis and fatty liver show lower expression levels of genes related to glycolysis, gluconeogenesis, and tricarboxylic acid (TCA) cycle, especially oxidative phosphorylation, protein ubiquitination, and ubiquinone synthesis, but have predominant activation of selenoamino acid metabolism, ribosome and replication, and repair [124,125]. The expression level of *FGF21* and *APOBR* in the liver are tightly related to NEB condition, and ketosis becomes a potential biomarker [123,126,127]. Furthermore, Soares et al. [128] found that *PPARA* and *ACACA* also have varying expression levels in different metabolic conditions, and there are 24 ketosis-related SNPs located in seven chromosomes. The pathogenesis of those diseases is similar to the non-alcoholic fatty liver disease (NAFLD) in humans. A study found that knock-downed *Fasn*, *Thrsp*, *Pklr*, and *Chchd6* could alleviate steatosis and insulin resistance in mice with NAFLD by downregulating mitochondrial respiration, indicating mitochondria dysfunction might be the key to NAFLD [129]. While Xu et al. [130] found that the plasma concentrations of neurosecretory protein FGA, c1 inhibitor (C1INH), serum amyloid A(SAA), transthyretin (TTR), hepcidin, apoprotein C III (APoCIII), amyloid precursor protein (APP), cystatin C (CysC), osteopontin (OpN) are significantly decreased in cows with fatty liver, not only proved the mitochondria dysfunction of NAFLD. Not only BHBA, the golden standard for ketosis diagnosis, is one of the most altered components in the metabolome profile of ketosis cows [48], but also serum 4-hydroxy-6-methyl-2-pyrone and cinnamoyl glycine show their potential as ketosis biomarkers [131]. Ketosis and fatty liver are high susceptive diseases for transition dairy animals and similar to NAFLD, which have been widely researched by omics methods. With the references of human studies, multi-omics studies for these diseases may produce great progress.

Mastitis, inflammation of the mammary gland, is the most common and costly disease of dairy cattle, which could induce by breast injury, environmental microorganisms (*Enterobacteriaceae*, *Streptococcus* spp., *Lactococcus* spp., *Prototheca* spp., etc.), and contagious pathogens (*Streptococcus lactis*, *Streptococcus agalactiae*, and *Staphylococcus aureus*). There are two types of mastitis, clinical or subclinical, depending on milk SCC and properties [132,133,134]. Clinical mastitis has symptoms including redness and swelling udder, decreased milk yield and quality with an SCC higher than 500,000 cells/mL (or 400,000 cells/mL in Europe), while subclinical mastitis lacks diagnosis signs in the milk or udder [135]. Research using omics has made a great progress in screening diagnose indices for subclinical mastitis. Thomas et al. [34,57,59] integrated metabolomics, peptidomics, and proteomics to investigate *Streptococcus uberis* mastitis and found that top abundance proteins change from caseins, β-lactoglobulin, and α-lactalbumin to albumin, lactoferrin, and IgG after challenge, and acute-phase protein (APP), mammary-associated serum amyloid A 3 (M-SAA3), haptoglobin, and C-reaction protein could be potential infectious mastitis indicators. At the metabolites level, the carbohydrates and nucleic acid concentration in milk dropped significantly in the acute phase. In contrast, lipid metabolites and peptides levels, especially the bile acid-nuclear receptor FXR signaling pathway, have been significantly elevated in the *Streptococcus uberis* challenged cows. In addition, *S. aureus* and *E. coli* mastitis have different cytokines reactions and lead to varying severity [134], indicating bacterial mastitis might have different biomarkers corresponding to Gram-positive and -negative pathogens or different pathogenesis pathways, which still need revealing.

Subacute ruminal acidosis (SARA), a metabolic disease mainly caused by feeding rumen microbiota fermentable carbohydrates (like corn and wheat) highly and induced consequent accumulation of organic acids, affects cows behavior, rumen fermentation, and metabolism, leading to systemic symptoms [136]. Most research about SARA aimed to detect specific indices or the change of microorganisms but lacks screening and analysis of the pathogenesis with a broader scope [137]. In recent years, more cognition about the SARA mechanism has been brought by omics techniques. Single-omics studies revealed changes in composition, transcription, and metabolites of rumen microbiota but still could not explain the mechanism of SARA-induced production decrease [30,138]. An in vitro study of Murovec et al. [41] showed that the inhibited fermentation reactor has an altered abundance of acetate, caprylate, trimethylamine, thymine, pyruvate, alanine, xanthine, and succinate. Zhang [32] combined rumen microbiome, metabolomics, epithelial genomic, and milk microbiota analysis, and found that SARA could disturb normal ruminal symbiotic flora and biosynthesis, especially valine, leucine, and isoleucine synthesis pathways (*p* < 0.05), elevating the level of toxic and proinflammatory bacterial metabolites (*p* < 0.05), meanwhile, the expression level of proinflammatory cytokines (IL-1β, IL-2, IL-22, etc.) increased (*p* < 0.05) and anti-inflammatory(IL-6) ones decreased(*p* < 0.05), in addition, SARA will increase milk somatic cell count (SCC) with a dropped milk protein and lipid component (*p* < 0.05). Li et al. [28,29] found that young calves with high starch induced rumen acidosis performs a different rumen epithelial transcriptome and meta-trascriptome profile with correlated rumen microbiome, liver transcriptome pattern also involved in this process, the abundance of *Olsenella*, *Desulfovibri*, and *Fusobacterium necrophorum* increased significantly in the rumen fluid, and 95 genes in the liver changed with differed microbiome rRNA expression, among them, 77 genes are enriched in the pathways of membrane-bounded organelle and transferase activity. Six-hundred-and-seventy-two epithelial genes related to cell signaling and morphogenesis showed significantly altered expression, in which, 12 genes (*COX5B*, *KRT78*, *KRT15*, *ATP5I*, *ATP5L*, *ATP5G2*, *COX8B*, *COX8A*, *UBC*, *DSP*, *ITM2B*, and *C10H15orf48*) related to hydrogen ion transmembrane transport only exists in the acidosis group; other differentially expressed genes are mostly involved in cell division and growth, like membrane-bounded organelle, cytoplasm, cellular component organization or biogenesis.

Based on clinical studies, omics analysis could give researchers a systematic cognition of certain diseases and evaluate the mechanism of clinical manifestations in different organs and levels, enhancing the knowledge about how diseases affect dairy production for more specific diagnosis and therapy. Although diary research is relatively hard to have a large quantity of biology replication to ensure the statistically robust and overcome obstacles in distinguishing pathology and physiology process, omics-based research on dairy veterinary still has increased contributions to the knowledge about the pathogenesis and corresponding genetic information of diseases in recent years. Multi-omics could combine the data from multiple organs and levels to demonstrate a complete diagram of diseases, clarifying the response of the rumen–liver–mammary gland network to pathogenetic factors, providing more effective solutions for prevention, diagnosis, and treatments.

## 6. Conclusions and Prospects

This review summarized recent genomics, transcriptomics, proteomics, and metabolomics research about physiology, feeding and management, and veterinary in dairy animals. In researching dairy animals, we must remind ourselves that the physiology and etiology are complicated phenomena based on heritable and acquired traits in multiple organs and levels. Those factors may be relatively isolated and work as independent systems while they are connected and weaved a tangled web of regulation. Each factor that affects any organ level may have a systematic influence, some of them are physiological responses, and others may become a part of the pathological process. From feed intake to milk yield and components, only metabolites themselves need to pass through multiple organs, while more regulation processes are involved in transcription and expression levels, needless to say, the complicated rumen fermentation. In dairy research, investigations on production performance show the effectiveness of factors; studies on certain levels and indices indicate the mechanism of factors; omics studies would reveal the response of the organs to factors; multi-omics research would connect them, demonstrate how the factor, directly and indirectly, affects the body, and how the body reacts to the factor. Because the ruminal fermentation mechanism is still not completely discovered, non-targeted proteomics and metabolomics have provided researchers with a systematic scope on crucial genes, proteins, and metabolites that regulates the metabolic pathways and the mechanisms of breeding selection, nutritional management, and diseases prevention. However, there are still limits on the single omics to combine multiple levels and organs and build a panoramic view of certain factors’ impact on the dairy animals and tracking the mechanism of its impact on their performance. With the assistance of multi-omics methods, researchers could screen more genes related to heritable traits, clarifying mechanisms of lactation physiology as well as the pathology of metabolic diseases. These promising methods would draw all organs and levels together to construct a whole vision of dairy production and establish novel directions for dairy research.

## Figures and Tables

**Table 1:** Omics applied in the dairy research.

First Author	Omics Applied	Year	Techniques	Targeted/Non-Targeted	Outcome	Reference
Pryce	Genomics	2014	-	-	Residual feed intake could be used as a breeding trait	[14]
Sigdel	Whole Genomic Mapping	2019			At least three different genomic regions on BTA5, BTA14, and BTA15 are strongly associated with milk production under heat stress	[15]
Tarekegn	Genomics	2021	-	-	Fertility-involved SNPs are different in Swedish red and Holstein cows	[16]
Feugang	Spermatozoa transcriptomics	2010	-	-	CD36 molecule decreased in low fertility bulls	[17]
Canovas	Milk transcriptomic	2010	-	-	Over 33,000 SNPs involved in lactation process	[18]
Akbar	Liver transcriptomic	2013	-	-	Feed restriction but not L-carnitine increased expression of GPX3,PC, PDK4, SAA3, and ADIPOR2	[19]
Fagerlind	Spermatozoa transcriptomics	2015	-	-	Mir-502-5p, mir-1249, mir-320a, mir-34c-3p, mir-19b-3p, mir-27a-5p and mir-148b-3p expressed differently with fertility	[20]
Wang	miRNA Transcriptomic	2016	-	-	MiRNAs expressed in these five tissues play roles in regulating the transportation of AA for downstream milk production	[21]
Li	Rumen Meta transcriptomics	2017	-	-	Carbohydrate active enzymes are related to feed efficiency	[22]
Comtet-Marre	Rumen Meta transcriptomics	2017	-	-	Cellobiose-phosphorylase, amylase, hemicellulases, cellulases, pectinase, and oligosaccharidases are main carbohydrate active enzymes	[23]
Song	Hind gut microbiome and meta-transcriptomic	2017	454 sequencing	-	SCFA alters the hindgut microbiome and their transcripts	[24]
Wang	Rumen Transcriptomic	2017	-	-	Expression of proliferation and apoptotic processes (BAG3, HLA-DQA1, and UGT2B17) related protein changes with different forages	[25]
Sollinger	Rumen meta transcriptomic	2018	-	-	Methyl-reducing but not CO_2_-reducing methanogens were positively correlated with methane emissions. Methanosphaera is the dominating methanol-reducing methanogen.	[26]
Putz	miRNA Transcriptomic	2019	-	-	46 miRNAs changes in the transition period	[27]
Li	Rumen meta-transcriptomic, liver transcriptomic	2019	-	-	627 gene involved in cell signaling and morphogenesis expressed differentially during acidosis	[28]
Li	Rumen meta-transcriptomic. Rumen epithelial transcriptomic	2019	-	-	Acidosis affected the expression of lipid metabolism involved genes	[29]
Ogunade	Metatranscriptomic	2019	-	-	Carbohydrate, amino acid, energy, vitamin and co-factor metabolism pathways, and bacterial biofilm formation pathways changes in the ruminal acidosis	[30]
Ametaj	Rumen metabolomic	2010	^1^H-NMR, GC-MS	Non-targeted	Over 30% proportion of barley grain diet increased potentially toxic metabolites	[31]
Zhang	Metabolomic, transcriptomic	2015	GC-MS	Non-targeted	Ruminal xanthine, hypoxanthine and uracil, biogenic amines, ethanolamine, glutaric acid, and amino acids concentrations elevated in the acidosis	[32]
Forde	Follicular-fluid metabolomic	2016	GC-MS	Non-targeted	Follicular-fluid of dry cows have higher tyrosine, phenylalanine and valine and fatty acids heneicosanoic acid and docosahexaenoic acid concentrations	[33]
Thomas	Milk metabolomics	2016	LC-MS	Non-targeted	Metabolites relevant to carbohydrate and nucleotide decrease after infection	[34]
Alejandro	Ruminal microbiome & metabolomic	2016	LC-MS	Non-targeted	Vitamin E changes rumen microbiome and enhances dry matter degradation	[12,35]
Humer	Serum metabolomic	2016	LC-ESI	Non-targeted	Excessive sphingolipids and phospholipids degradation is related to decreased insulin sensitivity in transition cows	[36]
Sun	Urine metabolomic	2016	GC-TOF/MS	Non-targeted	Hippuric acid and N-methyl-glutamic concentrations are significantly different between alfalfa hay fed and corn stover fed cows	[37]
Dai	Milk transcriptomic and proteomic	2017	LC-MS	iTRAQ labelling	Rice stover inhibits protein synthesis of dairy cows	[38]
Artegoitia	Rumen Fluid Metabolomic	2017	LC-MS	Non-targeted	linoleic and alpha-linolenic metabolism are correlated to daily growth	[39]
Sun	Umbilical blood metabolomic	2017	^1^H-NMR	Non-targeted	Rumen-protected arginine supplementation altered metabolic pathways of amino acid, carbohydrate and energy, lipids and oxidative stress metabolism of pregnancy cows	[40]
Murovec	Metabolomics	2018	^1^H-NMR	Non-targeted	Simulated an in vitro acidosis rumen model	[41]
Elolimy	Fecal metabolomic	2019	LC-MS	Non-targeted	Rumen-protected methionine supplementation onlate-pregnancy cows enhanced endogenous antibiotics synthesis, also hindgut functionality and health of their calves	[42]
Ogunade	ruminal fluid Metabolomics	2019	LC-MS	Non-targeted	Live yeast supplementation increased the concentrations of 4-cyclohexanedione and glucopyranoside and decreased the concentrations of threonic acid, xanthosine, deoxycholic acid, lauroyl carnitine, methoxybenzoic acid, and pentadecylbenzoic acid	[26]
Sun	Metabolomic, transcriptomic,	2020	GC	Non-targeted	Propionate, glucose, and amino acid concentration decreased in feeding with low-quality corp. Hippuric acid is the biomarker of corn stover fed cow	[43]
Zhang	rumen fluid metabolomic	2020	LC-MS	Non-targeted	Metabolites involved in protein digestion and absorption, ABC transporters, and unsaturated fatty acid biosynthesis pathways are correlated with milk yield	[44]
Clemmons	Rumen Fluid Metabolomic	2020	LC-MS	Non-targeted	Metabolites involved in amino acid and lipid metabolism are related to feeding efficiency	[45]
Xue	Rumen Metagenomics and meta-metabolomics	2020	GC-MS	Non-targeted	Rumen microbial composition, functions, and metabolites, and the serum metabolites are contributed to milk protein yield	[9]
Ogunade	Ruminal microbiome &metabolomic	2020	LC-MS	Non-targeted	DFMs alter rumen metabolites pattern and microbiome	[46]
Wang	Serum metabolomic	2020	GC−TOF/MS	Non-targeted	Rumen-Protected Betaine alters arginine synthesis and proline degradation and cyanoamino acid synthesis, promotes milk production	[47]
Luke	Serum metabolomic	2020	^1^H-NMR	Non-targeted	Quantified the relationship between NMR spectra and concentrations of the current gold standard serum biomarker of energy balance, beta-hydroxybutyrate	[48]
Lisuzzo	Serum metabolomic	2022	^1^H-NMR	Non-targeted	Correlations between serum ketone levels and milk lipid components in cows	[49]
Wang	Milk and rumen metabolomic	2021	UPLC-qTOF-MS	Non-targeted	Supplementation of perilla frutescens leaf could alter the ruminal metabolic profiles and milk synthesis through regulation of the pathways of pyrimidine metabolism and biosynthesis of unsaturated fatty acids	[50]
Gu	Milk Transcriptomic Metabolomic	2021	LC-MS/MS	Non-targeted	Rumen-protected methionine supplement increased α-ketoglutaric acid concentration, and related to rumen Thermoactinomyces, Asteroleplasma and Saccharofermentan abundance	[8]
Stergiadis	Rumen lipidomic, metabolomics, and microbiome	2021	GC (lipidomic), NMR (metabolomic)	Non-targeted	Cows with high milk fatty acid have higher butyrate, propionate and tyrosine and lower concentrations of xanthine and hypoxanthine concentrations	[51]
Wang	Rumen microbiome & metabolomic	2021	UPLC-QTOF/MS	Non-targeted	Rumen-protected glucose increased bacterial richness and diversity, also acetate, propionate, butyrate, and total volatile fatty acid in the rumen	[52]
Peddinti	Spermatozoa Proteomics	2008	DDF-2-LC-MS	Non-targeted	High-fertility bull and higher protein expression in energy metabolism, cell communication, spermatogenesis, and cell motility	[53]
Ledgard	Uterine luminal proteomics	2012	2-DE-MS	Non-targeted	Phosphoserine aminotransferase 1, purine nucleoside phosphorylase, and aldose reductase expression are related to the embryo growth environment	[54]
Saadi	Sperm proteomics	2013	LC-MS/MS	Non-targeted	Proteins involved in sperm capacitation, sperm–egg interaction, and sperm cytoskeletal structure were decreased in pyriform sperm, whereas proteins regulating antioxidant activity, apoptosis, and metabolic activity increased	[55]
Li	Milk proteomic	2015	2-DE- MALDI-TOF/TOF-MS		Process method of corn influences milk proteome pattern	[56]
Thomas	Milk peptidomics	2016	LC-MS/MS	Non--targeted	The abundance of caseins, beta-lactoglobulin, and alpha-lactalbumin to albumin, lactoferrin, and IgG shifted during the infection	[57]
Zachut	follicular fluids proteomics	2016	LC-MS	Non-targeted	Protein relevant to follicular function expressed differently in less fertility cows	[58]
Mudaliar	Milk proteomics	2016	LC-MS	Non-targeted	Antimicrobial peptides concentration elevates in the acute phase of mastitis	[59]
Snelling	Rumen metaproteomic	2017	2-DE-LC-MS	Non-targeted	2D-PAGE reveals key structural proteins and enzymes in the rumen microbial community	[60]
Skibiel	Liver proteomics	2018	nano-UPLC	Non-targeted	Oxidative phosphorylation, mitochondrial dysfunction, farnesoid X receptor/retinoid X receptor (FXR/RXR) activation, and the methylmalonyl pathway changes in the heat stress	[61]
Veshkini	Liver proteomic	2020	LC-MS/MS	Non-targeted	EFA and CLA status in transition cows had an impact on energy, lipid and vitamin metabolisms, and oxidative stress balance	[62]
